# Computer-Assisted Evaluation of Zygomatic Fracture Outcomes: Case Series and Proposal of a Reproducible Workflow

**DOI:** 10.3390/tomography11020019

**Published:** 2025-02-18

**Authors:** Simone Benedetti, Andrea Frosolini, Flavia Cascino, Laura Viola Pignataro, Leonardo Franz, Gino Marioni, Guido Gabriele, Paolo Gennaro

**Affiliations:** 1 Maxillofacial Surgery Unit, Department of Medical Biotechnology, S. Maria alle Scotte University Hospital of Siena, 53100 Siena, Italy; simone.benedetti1992@gmail.com (S.B.); andreafrosolini@gmail.com (A.F.); flaviacascino@hotmail.com (F.C.); lauraviolap@gmail.com (L.V.P.); guido.gabriele@unisi.it (G.G.); paolo.gennaro@unisi.it (P.G.); 2 Phoniatrics and Audiology Unit, Department of Neuroscience DNS, Univeristy of Padova, 31100 Treviso, Italy; leonardo.franz@unipd.it

**Keywords:** trauma, zygomatic fracture, surgical outcome, computer-assisted surgery (CAS), mirroring

## Abstract

Background: Zygomatico-maxillary complex (ZMC) fractures are prevalent facial injuries with significant functional and aesthetic implications. Computer-assisted surgery (CAS) offers precise surgical planning and outcome evaluation. The study aimed to evaluate the application of CAS in the analysis of ZMC fracture outcomes and to propose a reproducible workflow for surgical outcome assessment using cephalometric landmarks. Methods: A retrospective cohort study was conducted on 16 patients treated for unilateral ZMC fractures at the Maxillofacial Surgery Unit of Siena University Hospital (2017–2024). Inclusion criteria included ZMC fractures classified as Zingg B or C, treated via open reduction and internal fixation (ORIF). Pre- and post-operative CT scans were processed for two- and three-dimensional analyses. Discrepancies between CAS-optimized reduction and achieved surgical outcomes were quantified using cephalometric landmarks and volumetric assessments. Results: Out of the 16 patients (69% male, mean age 48.1 years), fractures were predominantly on the right side (81%). CAS comparison between the post-operative and the contralateral side revealed significant asymmetries along the X and Y axes, particularly in the fronto-zygomatic suture (FZS), zygo-maxillary point (MP), and zygo-temporal point (ZT). Computer-assisted comparison between the post-operative and the CAS-simulated reductions showed statistical differences along all three orthonormal axes, highlighting the challenges in achieving ideal symmetry despite advanced surgical techniques. CAS-optimized reductions demonstrated measurable improvements compared to traditional methods, underscoring their utility in outcome evaluation. Conclusions: CAS technology enhances the precision of ZMC fracture outcome evaluation, allowing for detailed comparison between surgical outcomes and virtual simulations. Its application underscores the potential for improved surgical planning and execution, especially in complex cases. Future studies should focus on expanding sample size, refining workflows, and integrating artificial intelligence to automate processes for broader clinical applicability.

## 1. Introduction

Zygomatico-maxillary complex (ZMC) fractures are commonly encountered in maxillofacial surgery practice, accounting for approximately 24% of all facial trauma cases [1]. Injuries leading to ZMC fractures may typically result from physical assaults, falls, road traffic accidents, and sports-related injuries [2,3]. High-energy trauma may cause comminuted ZMC fractures, resulting in secondary morphological disfigurement. In fact, the zygomatic bone plays a critical role in facial aesthetics and function, determining midfacial width and protrusion, contributing to the contour of the midface, and protecting the orbital contents [4,5]. Displacement of this bone can lead to facial asymmetry and ophthalmic symptoms, including restricted ocular motility, diplopia, exophthalmos, and enophthalmos [2,6,7]. Based on this, the importance of an accurate reduction and stabilization of ZMC fractures may be easily understood.

Traditional methods of treating ZMC fractures have focused on either closed or open reduction techniques, with or without internal fixation. Open reduction with internal fixation (ORIF) is the gold standard for treating unstable fractures, allowing for direct visualization of fracture lines and the placement of fixation devices [2,5]. However, achieving precise anatomical reduction remains challenging, particularly in cases of comminuted fractures [8].

Computed tomography (CT) imaging plays a crucial role in the management of ZMC fractures, providing detailed visualization of bone anatomy, fracture patterns, and associated injuries, allowing simultaneous volumetric 3D rendering of the involved area. As a result, the most commonly used diagnostic system for the diagnosis and classification of ZMC fractures (namely the Zingg classification [3]) is based on high-resolution CT scan findings. At the same time, post-operative CT data are crucial for evaluating surgical outcomes.

Along with the evolution of CT technology, the computer-assisted surgery (CAS) paradigm has progressively spread into the cranio-facial surgery field [9,10], with the most extensive body of evidence found in zygomatic implant placement for dental rehabilitation [11]. CAS refers to the use of advanced technologies, such as surgical navigation, computer-aided design (CAD), and computer-aided manufacturing (CAM), to enhance surgical planning, operating technique, and outcome evaluation. CAS enables the integration of patient-specific imaging data to create three-dimensional (3D) models, which allow surgeons to simulate optimal fracture reduction and assess outcomes with unparalleled precision [12]. This technology has shown promise in improving the accuracy of fracture realignment and facilitating reproducible results, especially in complex cases where traditional methods may fall short [4]. Despite this growing adoption, its application in maxillofacial traumatology, particularly in ZMC fractures, remains inconsistent [13]. Standardized workflows for its use are lacking, and its implementation is often confined to clinical research settings or secondary interventions rather than routine practice in most maxillofacial surgery units [14]. By bridging this gap, CAS could offer a transformative tool for achieving better anatomical reduction and symmetry in the routine treatment of ZMC fractures, ultimately addressing the limitations of traditional freehand techniques [15].

In the present investigation, we aimed to evaluate the feasibility of CAS application in assessing the outcomes of zygomatic fracture reduction using a cephalometric coordinates system. As a secondary aim, we evaluated the surgical outcome, comparing the actual post-operative result with an ideal virtual planning obtained from CAS utilization.

## 2. Materials and Methods

### 2.1. Study Design and Ethical Approval

To address the research purposes, we performed a retrospective cohort study including patients treated by the Maxillo-Facial Surgery Unit of the University Hospital “Le Scotte” in Siena, Italy, between January 2017 and June 2024. The study protocol was designed in conformity with the ethical guidelines of the 1975 Declaration of Helsinki and was approved by the Ethical Committee for Clinical Research of the University Hospital of Siena (approval no. 18/2023). All data have been reported according to the STROBE (STrengthening the Reporting of OBservational studies in Epidemiology) guidelines (www.strobe-statement.org). The study included patients who were diagnosed with ZMC fractures and brought to the attention of the Surgical Unit, undergoing surgery between 1 January 2017 and 30 June 2024.

### 2.2. Inclusion/Exclusion Criteria and Data Collection

Inclusion criteria for the study were the following: (i) adult patients, (ii) ZMC fractures classified as type B or C according to Zingg classification, and (iii) patients treated with surgical ORIF of the fractures [3]. Some patients were excluded from the study for the following reasons: (i) bilateral ZMC fractures; (ii) presence of other concomitant midface fractures; (iii) previous midface fractures; (iv) incomplete radiological, surgical, or follow-up data.

The collected data for each patient included the following: (i) personal data, (ii) patient’s history (trauma dynamics, past medical history, occupational background, present and past use of medication), (iii) clinical characteristics of the fracture (classification of the patient according to Zingg [3], (iv) surgical data (number of accesses, methods of reduction and fixation), (v) pre-operative and post-operative CT scan data, (vi) clinical follow-up data.

### 2.3. Surgical Approaches

All the surgeries were performed under general anesthesia by the same Maxillo-Facial surgeons team. For each patient, either two (infraorbital/transconjunctival + lateral orbital approach) or three (infraorbital/transconjunctival + lateral orbital + intraoral approach) surgical approaches were performed to expose the fractures, depending on the entity of the zygomatic displacement, the number of bone fragments and the surgeon’s preference [16].

### 2.4. Anatomical Landmarks

**Figure 1:**
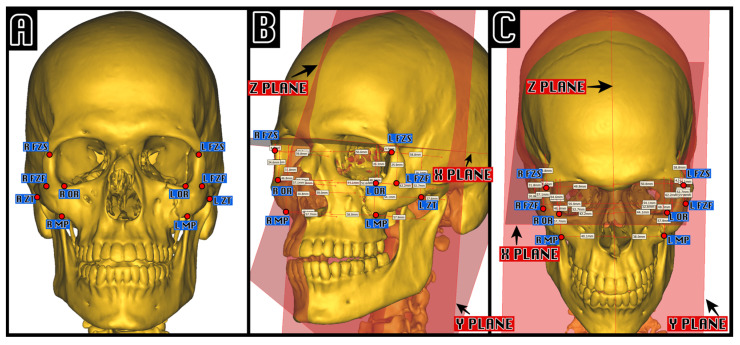
Example of cephalometric analysis in a computer-assisted virtual left ZMC fracture. On the left (**A**), the five cephalometric zygomatic points, proposed by Giran, are shown: FZF, foramen of the zygomaticofacial nerve; FZS, zygomaticofrontal suture; MP, zygo-maxillar point; ZT, zygo-temporal inferior; OR, orbitale. On the center and on the right is an example of visual cephalometric analysis visualization in an oblique projection (**B**) and in a Worms-Bretton projection (**C**), highlighting the measurements between each point and the three orthonormal planes X, Y, and Z.

In order to compare the position of the zygomatic bone fragments before and after surgery and comparing them to the virtual computer-assisted reduction, five anatomical zygomatic landmarks proposed by Giran [2] were adopted and marked (Figure 1), as well as the orthonormal coordinate system, which was constructed as follows:

- The Z median plane passing through the midpoint of the fronto-nasal suture (MidM), the midpoint of the posterior clinoid process (MidClp), and the foramen caecum (Fc).
- The X-plane, perpendicular to the Z-plane, and passing through MidM and MidClp.
- The Y-plane, constructed perpendicular to Z and X, and passing through MidClp.

For each landmark point, the distance between itself and the three orthogonal planes XYZ was measured and compared between pre-operative, post-operative, and computer-assisted zygomatic positions.

### 2.5. Digital Workflow

**Figure 2:**
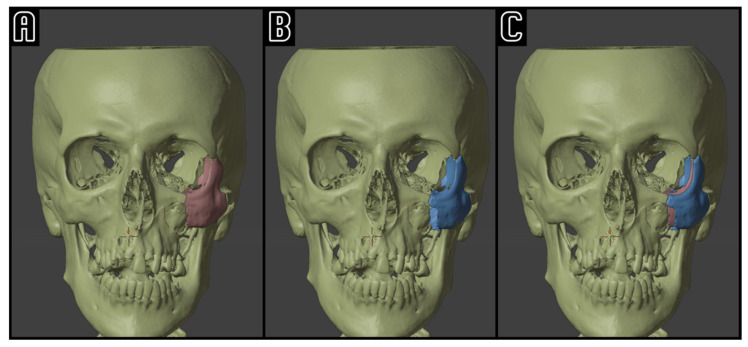
Example of computer-assisted reposition simulation: on the left side (**A**), the pre-operative situation with a conspicuously dislocated ZMC (red); on the center (**B**), the ZMC (blue) is virtually repositioned. On the right side (**C**), the superimposition of the two ZMC positions, highlighting the simulated symmetrization of the midface.

**Figure 3:**
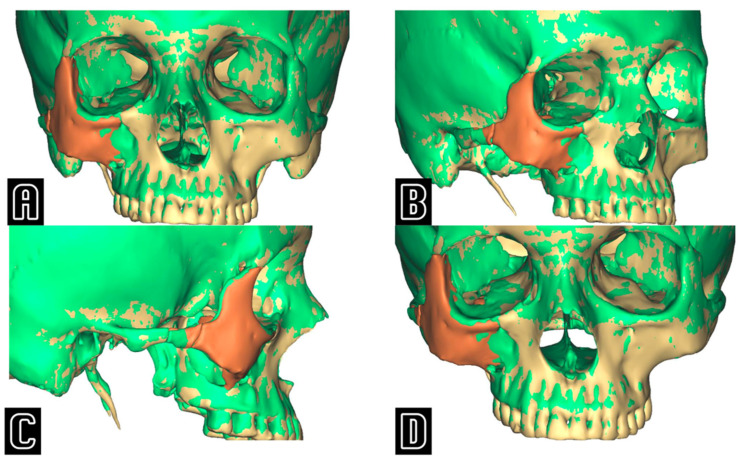
Frontal (**A**), oblique (**B**), lateral (**C**), and downward inclined (**D**) projections showing the superimposition between the actual post-operative result (green) and the virtually simulated position of the zygoma (in beige the skull, in orange the repositioned zygoma), emphasizing the undercorrection of the post-operative ZMC when compared to the computer-assisted “optimal” position.

For each patient, the workflow was the following: (1) pre-operative and post-operative CT scan acquisition; (2) definition of Hounsfield range of interest [17] and CT scan segmentation using Mimics inPrint Software version 3.0 (Materialise N.V., Leuven, Belgium) (Figure 2); (3) bone fragments isolation using the split tool; (4) mirroring of the contralateral healthy side; (5) definition of zygomatic anatomical landmarks and orbital volume measurement; (6) computer-assisted optimal reposition simulation (Figure 2); (7) anatomical landmark data extraction and volumetric analysis.

### 2.6. Statistical Analysis

Descriptive statistics, including mean, median, standard deviation (SD), and standard error (SE), were calculated for each anatomical landmark and its respective deviation along the X, Y, and Z axes. The Shapiro–Wilk test was used to assess the normality of data distributions. Paired sample comparisons between the fractured and contralateral (healthy) sides, as well as between post-operative and computer-assisted surgery (CAS)-simulated reductions, were performed using the Wilcoxon signed-rank test for non-parametric data. The statistical analyses were performed using Jamovi software (version 1.6, 2021, open access software available at https://www.jamovi.org, accessed on 24 November 2022).

## 3. Results

### 3.1. Study Population

Sixteen patients with a surgical ZMC fracture were included in the study. Individual patients’ data are summarized in Table 1. Of the included patients, 11 (69%) were men. The average age at the time of the surgical procedure was 48.1 ± 17.6 years. We found a predominance of the affected side being on the right malar bone: 13 cases (81%) vs. 3 (19%). Traffic accidents and accidental falls (three cases, 19%, both) were the most frequent etiologies, followed by sports-related injuries (two cases, 12.5%), with other causes aggregated in three patients (19%). No data were found in five cases (31%). Fractures were classified as Zingg type B (10 patients, 62%) or type C (6 patients, 38%), with a pair distribution of surgical accesses (50% two accesses and 50% three accesses). Nine patients (56%) were treated with 2-point fixation, while seven (44%) had three or more-point fixation methods. The mean follow-up period was 3.8 months (SD: 2.76).

For each patient, the digital workflow shown in the Methods section was applied, requiring approximately 90 min for patients classified as Zingg B and 105 min for Zingg C patients to complete the whole procedure and extract the cephalometric data.

### 3.2. Post-Operative Outcomes: Right-Left Discrepancy and Surgical Correction Versus CAS Optimal Reduction

Post-surgical cephalometric landmarks analysis on the three axes is reported in Table 2. Paired sample comparisons between the fractured and contralateral (healthy) sides showed no difference along the *Z*-axis, while in the *X*-axis results, only FZS (*p* = 0.017) indicated residual discrepancies in alignment. On the *Y*-axis, significant asymmetries were observed for MP (*p* = 0.009), FZF (*p* = 0.004), and ZT (*p* = 0.003). All the comparisons are reported in Table 2.

Comparisons between post-operative and CAS-simulated reductions along the *Z*-axis showed statistical significance for FZS (*p* = 0.002) and MP (*p* = 0.044), indicating measurable differences between surgical reduction and CAS simulation for these landmarks. *X*-axis discrepancies highlighted notable deviations still for FZS (*p* = 0.010) and MP (*p* = 0.020). For the *Y*-axis, FZS (*p* = 0.019), FZF (*p* = 0.011), and ZT (*p* = 0.025) indicated significant differences. All the comparisons are reported in Table 3.

Among the five anatomical landmarks analyzed across the three planes and two comparisons, the fronto-zygomatic suture (FZS) was the most frequently significant, showing discrepancies in four out of six analyses (Z, X, and Y axes for both CAS and contralateral side comparisons). The fronto-zygomatic foramen (FZF) and maxillary process (MP) were significant in three out of six analyses, particularly along the Y axis for both CAS and contralateral comparisons and the Z axis for CAS. The zygomatic tubercle (ZT) was significant in two out of six analyses, primarily along the Y axis. In contrast, the orbital rim (OR) showed no significant discrepancies in any comparison, suggesting consistent alignment.

Considering the five different anatomical landmarks across the three planes and two types of analysis, we found the following characteristics: FZS: significant in four out of six analyses (discrepancies on the Z, X, and Y axes with both CAS and contralateral sides); FZF: significant in three out of six analyses (discrepancies on the Y axis with both CAS and contralateral sides, and discrepancies on the Z axis with CAS); MP: significant in three out of six analyses (discrepancies on the Z axis with CAS and discrepancies on the Y axis with contralateral sides); ZT: significant in two out of six analyses (discrepancies on the Y axis with both CAS and contralateral sides); OR: never significant in any axis or comparison.

## 4. Discussion

This study analyzed a cohort of 16 patients undergoing surgical treatment for unilateral ZMC fractures. A male predominance (69%) and a higher prevalence of right-sided fractures (81%) were observed, consistent with the literature on ZMC fractures, which often were due to high-energy trauma (the etiologies of fractures in this study included traffic accidents, accidental falls, and sports-related injuries, with some cases lacking specific data). The application of a digital workflow requiring approximately 90–105 min, depending on fracture complexity, demonstrated the feasibility of integrating CAS into routine clinical practice. The use of CAS facilitated cephalometric measurements and enabled a detailed assessment of post-operative outcomes in relation to the contralateral side and CAS-optimized reduction. Significant right-left discrepancies were identified in the *X*-axis (FZS) and the *Y*-axis (MP, FZF, and ZT). While the lack of 25% of data (four cases) for the *Y*-axis needs to be stressed, these results suggest that achieving perfect symmetry remains challenging in certain regions, even with advanced surgical techniques. Similarly, comparisons between surgical outcomes and CAS-optimized reductions revealed significant discrepancies for at least two landmarks in each plane, highlighting areas where surgical accuracy could improve (Figure 3).

In our analysis, FZS was the most frequently significant landmark, highlighting challenges in achieving midfacial symmetry. FZF, MP, and ZT also showed significant discrepancies in multiple axes, which need to be underscored, considering their importance in both aesthetic and functional outcomes. The findings confirmed the need for enhanced precision in these regions, particularly for deeper or lateral landmarks like MP and ZT, where traditional methods often fall short. Conversely, OR (likely benefiting from its straightforward intra-operative exposure and direct fixation options) consistently showed no significant discrepancies, suggesting that conventional surgical techniques reliably address its alignment.

The optimal number of fixation points for ZMC fractures remains another subject of ongoing debate [5]. In this study, the fixation points varied depending on the fracture complexity and surgeon preference, with most cases employing two or three-point fixation techniques. Preliminary findings from our analysis suggest that the number of fixation points may influence post-operative symmetry, particularly in regions with greater complexity, such as the infraorbital rim or zygomatic arch. While three-point fixation is often recommended for providing enhanced stability and reducing rotational deformities, two-point fixation can be effective in cases with minimal displacement or simpler fracture patterns. However, discrepancies noted in deeper landmarks, such as MP and ZT, may indicate that additional fixation points could help achieve better alignment in certain cases. Further research is warranted to clarify the relationship between the number of fixation points and long-term functional and aesthetic outcomes. Prospective, controlled studies could provide more definitive guidance and help develop tailored approaches based on fracture characteristics and individual patient needs.

The findings of this study supported the growing body of evidence that CAS could enhance the evaluation and management of ZMC fractures. The ZMC is a critical structure in the midface, influencing midfacial width and projection. It has an irregular three-dimensional shape and a complex anatomical structure, forming the lateral wall of the orbit and being surrounded by various muscles. When ZMC fractures occur, the increased risk of functional and aesthetic defects complicates treatment. Thus, the main goal in treating ZMC fractures is to restore the midfacial contour, with the precise reduction being crucial; also, achieving successful reduction largely depends on the surgeon’s experience. The findings of this study support the growing body of evidence that CAS can enhance the pre-operative evaluation and management of ZMC fractures.

Previous studies supported the value of computer-assisted navigation systems in improving surgical precision and outcomes. For instance, Bao et al. highlighted the effectiveness of surgical navigation in restoring facial symmetry, particularly in complex fractures [1]. Similarly, He et al. reported that using surface markers during navigation-assisted surgery allowed for a highly accurate reduction in delayed fractures with minimal post-operative asymmetry [4]. In a recent article, Committeri et al. compared the performance in the management of patients with ZMC fractures treated using computer-assisted planning and traditional management. Their results showed that CAS reduced surgical time and post-operative complications but, most importantly, allowed greater intra-operative accuracy [18]. In addition, a newly released investigation by Hassan et al. [19] showed that CAS combined with 3D printing facilitates the anatomically accurate reduction and fixation of the ZMC fractures.

In the present study, the use of CAS technology allowed for objective comparisons between pre-operative planning and actual surgical outcomes. By analyzing both two-dimensional measurements and three-dimensional volumetric comparisons, this study suggested that CAS could provide detailed insights into the accuracy of fracture reduction. This technology not only facilitates the precise positioning of bone segments but also enables volumetric assessments that are essential for evaluating outcomes in complex fractures.

An important consideration in the evaluation of surgical outcomes using CAS technology is interobserver variability. Despite the standardized workflow employed in this study, differences in landmark identification and segmentation among evaluators may influence the reproducibility of results. This variability underscores the need for automated or semi-automated approaches to reduce subjective bias and improve consistency. For example, the integration of artificial intelligence for automatic landmark detection could standardize measurements and decrease operator dependency. A dedicated study investigating interobserver variability in the application of CAS technology would provide valuable insights into its reproducibility and help refine protocols to minimize potential inconsistencies. Addressing interobserver variability is essential to ensure that CAS is not only a precise but also a reproducible tool for assessing and improving outcomes in ZMC fracture management.

An additional application of the proposed computer-assisted workflow lies in the projection and customization of titanium mesh orbital implants required for orbital reconstructions. The use of CAS technology enables precise pre-operative planning and intraoperative execution, particularly in restoring orbital volume and contour. By integrating 3D imaging data, surgeons can accurately assess orbital defects and design patient-specific implants that ensure optimal anatomical fit and stability [20]. This approach is particularly beneficial in cases involving ZMC fractures with concomitant orbital wall involvement, where accurate restoration of the orbital framework is critical to avoid functional complications such as enophthalmos or diplopia. The ability to incorporate the projection of titanium mesh implants into the digital workflow further underscores the versatility of CAS in addressing complex midfacial fractures, offering both aesthetic and functional benefits. Future studies should explore the role of this workflow in improving outcomes for orbital reconstructions, particularly in challenging cases requiring extensive repair.

Despite the potential advantages of CAS, challenges remain. The time and cost associated with generating and working with 3D models may limit their widespread adoption in clinical practice, despite new technologies such as AI and deep learning models that could simplify and expand their applicability [21,22].

In this context, a study by Jiang et al. [23] underscored the potential of CAS. They used modified patient-specific surgical guides to address comminuted ZMC fracture reduction, highlighting that less-experienced surgeons can particularly benefit from CAS despite the high pre-operative effort and skills required. Moreover, as previously reported, there is still variability in the clinical outcomes depending on the surgeon’s experience and the complexity of the fracture [7]. Nonetheless, CAD-CAM technology represents a significant step forward in the pursuit of more predictable and reproducible outcomes in the treatment of ZMC fractures.

### Strengths, Limitations, and Future Directions

The limitations of this study are as follows: (i) the limited sample size (16 patients), which prompts caution in generalizing results; (ii) the exclusion of bilateral fractures or patients with complex midfacial injuries, thus limiting the applicability in more complex cases; (iii) the retrospective design, introducing possible information biases (including potentially limited data on etiology). Additionally, from a technical point of view, the reliance on manual segmentation and landmark identification could introduce inter-observer variability, even with a standardized workflow.

Despite these limitations, this study has several strengths, including the use of a reproducible and standardized digital workflow for outcome evaluation in ZMC fractures. The inclusion of patients with different degrees of fracture severity (Zingg type B and C) and fixation methods potentially improved the generalizability of the findings to diverse clinical scenarios.

Future research should aim to address these limitations by including a larger, more diverse cohort of patients and exploring the applicability of CAS in bilateral and comminuted fractures. A larger cohort of patients could also provide the opportunity to compare outcomes between groups who underwent different surgical approaches (e.g., number and type of accesses performed, number of plates used), offering valuable information to aid in the development of a symmetry-focused treatment algorithm for zygomatic fractures.

Prospective studies could enhance data consistency and further validate the reproducibility of the digital workflow. Advancements in artificial intelligence and machine learning could offer promising opportunities to automate landmark detection and segmentation, potentially reducing observer variability and improving efficiency. Moreover, long-term follow-up studies might assess the functional and aesthetic outcomes, providing a more comprehensive evaluation of CAS benefits. Integration of 3D printing and patient-specific surgical guides may further refine pre-operative planning and intraoperative execution, paving the way for more personalized and precise care in maxillofacial surgery.

## 5. Conclusions

This retrospective study highlights the significant potential of CAS in evaluating the outcomes of ZMC fracture treatment. The integration of CAS enabled precise comparisons between surgical results and optimized virtual reductions, revealing key discrepancies in critical cephalometric landmarks such as the fronto-zygomatic suture, zygo-maxillary point, and zygo-temporal point. These findings emphasize the challenges of achieving ideal symmetry using traditional surgical methods, even with advanced fixation techniques.

The standardized digital workflow employed in this study appeared to be reproducible and effective in enhancing the objectivity of outcome evaluation, supporting the adoption of CAS in routine clinical practice for zygomatic fractures. By facilitating both two-dimensional and three-dimensional analyses, CAS offers a valuable tool for surgeons to improve accuracy and achieve better functional and aesthetic outcomes. Despite its advantages, limitations such as high pre-operative time requirements, costs, and a small study cohort must be addressed. The study underscores the need for future research focusing on larger and more diverse patient populations, the inclusion of bilateral or comminuted fractures, and the use of artificial intelligence to streamline and automate processes. As CAS technology continues to evolve, its role in improving predictability, reproducibility, and precision in maxillofacial surgery is expected to expand, paving the way for more personalized and effective patient care.

## Figures and Tables

**Table 1:** Patients’ demographics and clinical data.

ID	Sex	Age (Years)	Trauma	Side	Zingg Classification	Associated Fractures	No. of Accesses	No. of Plates	Early Complications	Late Complications	Follow-Up (Months)
1	M	31	Sport	R	b	Orbital floor	3	3	None	None	2
2	M	55	MD	R	c	None	2	2	None	None	6
3	F	39	Car accident	R	b	None	3	3	V2 hypoesthesia	V2 hypoesthesia	6
4	F	68	Fall	R	c	Orbital floor	2	2	None	None	3
5	F	49	Syncopal episode	R	b	None	3	3	None	Sinusitis + plate removal	11
6	M	53	MD	R	b	Orbital floor, partial Le Fort I	2	2	MD	MD	0
7	M	50	MD	R	c	None	3	1 + 1 *	V1 hypoesthesia	Sinusitis	6
8	F	78	Fall	R	b	None	2	2	None	None	2
9	M	61	Horse kick	R	c	Mandibular angle	3	4	None	None	3
10	M	63	Fall	R	b	Orbital medial wall	2	2	V2 hypoesthesia,	MD	0
11	F	58	Car accident	R	b	Orbital floor	3	3	None	None	6
12	M	64	Bike fall	L	b	Orbital floor	2	2	V2 hypoesthesia	None	4
13	M	17	MD	R	b	Orbital floor,	2	2	None	None	1.5
14	M	30	MD	L	c	Orbital floor	2	2	V2 hypoesthesia	V2 hypoesthesia	4
15	M	28	Sport	R	c	Orbital floor	3	3	V2 hypoesthesia	V2 hypoesthesia	5
16	M	26	Car accident	L	c	Orbital floor	3	3	None	None	1

Abbreviations: M: male; F: female; MD: missing data; R: right; L: left; No.: number. * One plate is used for maxillo-zygomatic buttress, and one metallic wire is used for frontozygomatic suture.

**Table 2:** Comparison of post-operative anatomical discrepancies: post-operative fractured side vs. healthy side.

Measure	No.	Mean (mm)	Median (mm)	SD (mm)	SE (mm)	Normality (W)	Normality (*p*)	Statistic Test	Test (*p*)
Z-R-OR vs. Z-L-OR	16	40.506; 39.500	39.500; 39.500	5.802	1.450	0.713	<0.001	Wilcoxon W = 89.50	0.277
Z-R-MP vs. Z-L-MP	16	44.281; 44.150	44.150; 44.150	5.571	1.393	0.695	<0.001	Wilcoxon W = 89.50	0.277
Z-R-FZS vs. Z-L-FZS	16	49.019; 48.750	48.750; 48.750	1.941	0.485	0.974	0.896	Wilcoxon W = 46.50	0.277
Z-R-FZF vs. Z-L-FZF	16	48.900; 48.500	48.500; 48.500	1.633	0.408	0.880	0.039	Wilcoxon W = 43.50	0.214
Z-R-ZT vs. Z-L-ZT	16	61.075; 61.650	61.650; 61.650	3.297	0.824	0.865	0.023	Wilcoxon W = 35.00	0.164
X-R-OR vs. X-L-OR	16	24.663; 24.700	24.700; 24.700	5.892	1.473	0.652	<0.001	Wilcoxon W = 37.00	0.115
X-R-MP vs. X-L-MP	16	43.344; 45.150	45.150; 45.150	8.913	2.228	0.729	<0.001	Wilcoxon W = 49.00	0.339
X-R-FZS vs. X-L-FZS	16	2.013; 1.600	1.600; 1.600	1.652	0.413	0.917	0.149	Wilcoxon W = 14.00	0.017
X-R-FZF vs. X-L-FZF	16	27.050; 26.650	26.650; 26.650	4.120	1.030	0.963	0.710	Wilcoxon W = 64.00	0.842
X-R-ZT vs. X-L-ZT	13	30.769; 30.200	30.200; 30.200	4.849	1.345	0.836	0.019	Wilcoxon W = 39.00	0.685
Y-R-OR vs. Y-L-OR	12	55.133; 55.150	55.150; 55.150	3.376	0.975	0.958	0.758	Wilcoxon W = 43.00	0.398
Y-R-MP vs. Y-L-MP	12	45.775; 46.950	46.950; 46.950	7.115	2.054	0.953	0.680	Wilcoxon W = 63.00	0.009
Y-R-FZS vs. Y-L-FZS	12	51.267; 51.000	51.000; 51.000	6.347	1.832	0.555	<0.001	Wilcoxon W = 41.00	0.906
Y-R-FZF vs. Y-L-FZF	12	49.175; 49.600	49.600; 49.600	3.474	1.003	0.573	<0.001	Wilcoxon W = 76.00	0.004
Y-R-ZT vs. Y-L-ZT	12	20.725; 19.500	19.500; 19.500	6.039	1.743	0.968	0.883	Wilcoxon W = 78.00	0.003

Abbreviations: OR: orbital rim; MP: maxillary process; FZS: fronto-zygomatic suture; FZF: fronto-zygomatic foramen; ZT: zygomatic tubercle; No.: sample size; SD: standard deviation; SE: standard error; Normality (W): Shapiro–Wilk normality test statistic; Normality (*p*): *p*-value for normality test; Statistic Test: Wilcoxon signed-rank test statistic; Test (*p*): *p*-value for Wilcoxon signed-rank test.

**Table 3:** Comparison of post-operative anatomical discrepancies: DSC vs. DRL across zygomatic landmarks.

Measure	No.	Mean (mm)	Median (mm)	SD (mm)	SE (mm)	Normality (W)	Normality (*p*)	Statistic Test	Test (*p*)
DSC-Z-OR vs. DRL-Z-OR	15	1. 147; 0.900	0.900; 0.900	1. 163	0.300	0.931	0.287	Wilcoxon W = 20.00	0.080
DSC-Z-MP vs. DRL-Z-MP	16	1. 531; 1.400	1.400; 1.400	1. 173	0.293	0.801	0.003	Wilcoxon W = 24.00	** 0.044 **
DSC-Z-FZS vs. DRL-Z-FZS	16	0. 475; 0.500	0.500; 0.500	0. 404	0.101	0.884	0.044	Wilcoxon W = 5.00	** 0.002 **
DSC-Z-FZF vs. DRL-Z-FZF	16	0. 831; 0.600	0.600; 0.600	0. 812	0.203	0.979	0.957	Wilcoxon W = 44.00	0.378
DSC-Z-ZT vs. DRL-Z-ZT	16	1. 994; 1.700	1.700; 1.700	1. 216	0.304	0.921	0.178	Wilcoxon W = 28.00	0.409
DSC-X-OR vs. DRL-X-OR	15	1. 027; 0.800	0.800; 0.800	0. 843	0.218	0.915	0.160	Wilcoxon W = 47.00	0.754
DSC-X-MP vs. DRL-X-MP	15	0. 633; 0.600	0.600; 0.600	0. 641	0.166	0.906	0.118	Wilcoxon W = 15.00	** 0.020 **
DSC-X-FZS vs. DRL-X-FZS	15	0. 342; 0.300	0.300; 0.300	0. 420	0.108	0.881	0.048	Wilcoxon W = 9.00	** 0.010 **
DSC-X-FZF vs. DRL-X-FZF	15	1. 300; 1.350	1.350; 1.350	0. 946	0.236	0.869	0.026	Wilcoxon W = 29.50	0.158
DSC-X-ZT vs. DRL-X-ZT	13	1. 631; 1. 300	1.300; 1.300	1. 029	0.285	0.953	0.643	Wilcoxon W = 22.00	0.609
DSC-Y-OR vs. DRL-Y-OR	12	1. 058; 0.900	0.900; 0.900	0. 936	0.270	0.878	0.083	Wilcoxon W = 24.00	0.450
DSC-Y-MP vs. DRL-Y-MP	12	2. 025; 1.300	1.300; 1.300	1. 960	0.566	0.845	0.032	Wilcoxon W = 15.00	0.120
DSC-Y-FZS vs. DRL-Y-FZS	11	0. 845; 1. 000	1.000; 1.000	0. 607	0.183	0.944	0.564	Wilcoxon W = 4.00	** 0.019 **
DSC-Y-FZF vs. DRL-Y-FZF	11	1. 418; 1.000	1.000; 1.000	1. 242	0.375	0.918	0.304	Wilcoxon W = 4.00	** 0.011 **
DSC-Y-ZT vs. DRL-Y-ZT	12	1. 692; 1.300	1.300; 1.300	0. 969	0.280	0.888	0.112	Wilcoxon W = 10.00	** 0.025 **

Abbreviations: DSC: discrepancy surgical-computed assisted surgery; DRL: discrepancy right left side; OR: orbital rim; MP: maxillary process; FZS: fronto-zygomatic suture; FZF: fronto-zygomatic foramen; ZT: zygomatic tubercle; No.: sample size; SD: standard deviation; SE: standard error; Normality (W): Shapiro-Wilk normality test statistic; Normality (*p*): *p*-value for normality test; Statistic Test: Wilcoxon signed-rank test statistic; Test (*p*): *p*-value for Wilcoxon signed-rank test. The bold are present where the *p*-value is inferior to 0.05.

## Data Availability

The data presented in this study are available on request from the corresponding author.

## Abbreviations

CAS—computer-assisted surgery; ZMC—zygomatico-maxillary complex; ORIF—open reduction and internal fixation; CT—computed tomography; CAD—computer-aided design; CAM—computer-aided manufacturing; FZS—fronto-zygomatic suture; MP—zygo-maxillary point; ZT—zygo-temporale inferior point; FZF—fronto-zygomatic foramen; OR—orbitale point; XYZ—orthonormal coordinate planes; SD—standard deviation; SE—standard error; AI—artificial intelligence; MD—missing data.
